# A High Sensitivity Micro Format Chemiluminescence Enzyme Inhibition Assay for Determination of Hg(II)

**DOI:** 10.3390/s100706377

**Published:** 2010-06-28

**Authors:** Kanchanmala Deshpande, Rupesh K. Mishra, Sunil Bhand

**Affiliations:** Chemistry Group, Birla Institute of Technology and Science, Pilani-Goa Campus, Zuarinagar Goa, PIN 403726, India; E-Mails: kanchan@bits-goa.ac.in (K.D.); rupeshm@bits-goa.ac.in (R.K.M.)

**Keywords:** high throughput assay, miniaturization, Hg(II), alcohol oxidase, peroxidase, enzyme inhibition, chemiluminescence, water

## Abstract

A highly sensitive and specific enzyme inhibition assay based on alcohol oxidase (AlOx) and horseradish peroxidase (HRP) for determination of mercury Hg(II) in water samples has been presented. This article describes the optimization and miniaturization of an enzymatic assay using a chemiluminescence reaction. The analytical performance and detection limit for determination of Hg(II) was optimized in 96 well plates and further extended to 384 well plates with a 10-fold reduction in assay volume. Inhibition of the enzyme activity by dissolved Hg(II) was found to be linear in the range 5–500 pg·mL^−1^ with 3% CV in inter-batch assay. Due to miniaturization of assay in 384 well plates, Hg(II) was measurable as low as 1 pg·mL^−1^ within 15 min. About 10-fold more specificity of the developed assay for Hg(II) analysis was confirmed by challenging with interfering divalent metal ions such as cadmium Cd(II) and lead Pb(II). Using the proposed assay we could successfully demonstrate that in a composite mixture of Hg(II), Cd(II) and Pb(II), inhibition by each metal ion is significantly enhanced in the presence of the others. Applicability of the proposed assay for the determination of the Hg(II) in spiked drinking and sea water resulted in recoveries ranging from 100–110.52%.

## 1. Introduction

Hg(II) is a highly toxic element that is found both naturally and as an introduced contaminant in the environment. Hg(II) is widely distributed in the Earth’s crust, sea water, ground and rain water, and its toxic effects on biological systems through direct uptake as well as by accumulation in food chain are well known [1]. Average Hg(II) level in the atmosphere are 3–6 fold higher than the pre-industrial estimates [2]. Hg(II) pollution has always been the area of concern. History records several major cases of Hg(II) poisoning. Among them the infamous Mina Mata tragedy in 1956 where Hg(II) was dumped into the sea and residents of the Mina Mata bay, Japan area began coming down with a strange nervous disorder [3]. The persistent nature of Hg(II) and its ill effect for years is supported by recent report on high concentration of Hg(II) in the water of Bhopal, India [4]. Atmospheric deposition of Hg(II) contains three principal forms, although the major component is inorganic Hg as Hg(II). Due to its high toxicity World Health Organization has set a limit for Hg(II) in drinking water which is 0.001 μg·mL^−1^ for water quality monitoring purposes [5]. This necessitates extensive study of existing analytical methods, identifying the gap between them and developing alternative methods for simple and quick determination of Hg(II) in the environment at very low concentrations.

Several analytical methods such as atomic absorption spectrometry, liquid chromatography with inductively coupled plasma mass spectrometry and others have been developed for Hg(II) analysis [6]. These techniques, although very sensitive for Hg(II) analysis, require extensive sample pretreatment, they use large amounts of organic solvent and do not give toxicological information. Biosensors provide a good alternative to all these problems as a simple, rapid and cost effective tool for the analysis of Hg(II) in the environment [7]. The remarkable affinity of Hg(II) for amino acids and proteins can cause structural and functional changes which can be utilized in the development of bio-analytical techniques. For Hg(II) compounds the primary route responsible for their toxicity is depletion of glutathione and bonding to the sulfhydryl (-SH) groups of proteins [8]. Reported inhibition based Hg biosensors mainly exploit the properties of enzyme inhibition or micro-organism toxicity for Hg determination. Hg ions are known as effective inhibitors of the catalytic activities of various enzymes such as AlOx [9], butyrylcholinesterase (BuChE) [10], glucose oxidase (GOD) [11], peroxidase (HRP) [12–13], invertase [14,15], glycerol 3-phosphate oxidase [16] and urease [17–21]. Recently array based enzyme biosensors have been reported for screening various environmental pollutants, mainly heavy metals and pesticides. The urease enzyme has been extensively used as a model enzyme to elucidate the applicability of inhibition assays for Hg(II), where reported work has focused on assays with immobilized urease [20]. The detection limit of optical determination of Hg(II) by urease are reported to be as low as 1ng·mL^−1^ [19], whereas for free BuChE it is reported up to 6 μM [10]. Successful Hg(II) analysis using AlOx using flow injection analysis by a thermal technique is reported to have a detection limit as low as 5 ng·mL^−1^ [9]. The AlOx exhibit high affinity for primary alcohols [22], moreover we observed that it has high stability in free form and the capability to discriminate potentially competing inhibitors in samples of mixtures of metal ions. These remarkable features make AlOx a perfect choice for Hg(II) inhibition studies. Co-exposure to metal ions such as cadmium (Cd), lead (Pb) and Hg is very common in the environment. Therefore it is imperative that toxicity studies of these metals in combination be adequately pursued. Interference studies using Cd(II) and Pb(II) as model inhibitors were reported extensively in Hg(II) analysis [23,24]. Individual inhibition of AlOx by these ions gives the idea of their relative toxicity levels [25].

Recently, the optical transducer has replaced all existing transducers for high throughput analysis due to its high sensitivity and interference free nature. The detector/transducer is not in contact with the reaction mixture, thus minimizing chances of fouling and giving less false results. In an optical microplate assay, product formation can be measured directly without any prior analyte preconcentration step or a later purification step for product. It is reported that among the optical techniques, chemiluminescence can be used for heavy metal analysis as the instrumental setup is relatively simple, free from instrumental interferences and highly sensitive [26–28]. There is significant interest toward miniaturization of analytical systems, since it allows the handling of low volume samples, reduction in waste generation and reagent consumption with increased sample throughput [29,30]. The analyses using reduced amounts of chemicals will not only have a positive impact on the environment but also on the assay costs. The developed method was tested for Hg(II) analysis in real water samples; the increasing Hg(II) sea water pollution cases have attracted the attention of many researchers towards Hg(II) analysis in this medium [31–34]. Re-suspension and remobilization of heavy metals from the coastal sediments due to natural disaster such as tsunamis, is also one of the major problems today. Inorganic mercury as Hg(II) being the major form of Hg(II) in sea water, we have extended our study for Hg(II) analysis in sea water at ultra low concentrations.

The aim of this work was to develop a highly sensitive Hg(II) inhibition-based optical biosensor using free AlOx using a chemiluminescence technique. The assay has been demonstrated in 96 and 384 well plates for Hg(II) determination in drinking water at levels as low as 1 pg·mL^−1^. With miniaturized assays we could significantly reduce the consumption of the AlOx enzyme and Hg(II) and increase the assay throughput, thus ultimately improving assay economy. The assay presented can determine Hg(II) in the presence of Cd(II) and Pb(II) ions and non interfering threshold levels for Cd(II) and Pb (II) have been identified. The assay has been successfully extended for analysis of mixtures of Hg(II) along with Cd(II) and Pb(II). The inhibition patterns obtained are consistent with other reported studies using cell lines [25] and urease enzyme [19]. The developed method was applied to real samples such as drinking water and sea water samples with recoveries up to 110.52%. The sensitive determination of Hg(II) in sea water at levels as low as 5 pg·mL^−1^ is an important feature of our assay. The high storage stability at 4 °C of AlOx preloaded in micro wells over the period of 11 months was used as effective tool for high throughput Hg(II) determination.

## 2. Experimental

### 2.1. Enzymes and Reagents

Mercury AA/ICP calibration/check standard for environmental analysis, horseradish peroxidase (1.11.1.7) and alcohol oxidase (1.1.3.13) from *Pichia pastoris*, 5-amino-2,3-dihydro-1,4-phthalazinedione (luminol) were purchased from Sigma Chemical CO. (MO, USA), 4-Iodophenol was purchased from Aldrich (USA). Lead and Cadmium standard solution (999 ± 2 mg·L^−1^), methanol (99.9%), ethanol (99%), hydrogen peroxide (30%), sodium phosphate dibasic and sodium phosphate monobasic were from Merck (Germany). 1 mmol·L^−1^ luminol solution was prepared by dissolving 4 mg of luminol in 2 mL 0.1 M NaOH (Merck, Germany) and making up the volume to 20 mL by 0.1 M phosphate buffer (PB), pH 7.5. 1 mmol·L^−1^ of 4-iodophenol solution was also prepared freshly. All other reagents were of analytical reagent grade. Pyrogen free purified water (Milli-Q System, Millipore, USA) was used to prepare buffers. For chemiluminescence detection all the reagents were prepared in 0.1 M PB, pH 7.5. Working standards for all the reagents including inhibitor were prepared prior to analysis.

### 2.2. Apparatus and Equipment

Chemiluminescence measurements were recorded using a Multilabel Reader victor^3^ (Perkin Elmer, USA) instrument. Optiplate 96 microtiter plates (Nunc, Denmark), 384 well plates (Corning, USA) and micropipettes (Eppendorf, Germany) were also used. The pH, conductivity was measured using a model Seven multi digital pH and conductivity meter (Mettler Toledo, Switzerland). Six digit balance (Mettler Toledo, Switzerland) was used for weighing. A sonicator was used for degassing the phosphate buffer. A minicooler was used to reduce the temperature fluctuations of enzymes. For vortexing of solutions, a Spinix (Tarsons, India) was used.

### 2.3. Principle and Measurement Protocol

**Scheme 1.**
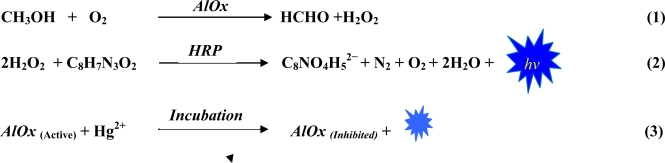
Representing bi -enzymatic (AlOx/HRP) reaction involved in the proposed assay.

The quantification of Hg(II) is done by inhibition which is due to the interaction of metal ion and the active site of an enzyme. Activity of free AlOx was monitored in the presence of HRP for inhibition studies using a chemiluminescence technique. During the interaction of exposed thiol or methylthiol groups of enzyme and metal ion, the metal ion decreases the catalytic activity of the enzyme. Chemiluminescent emission of the hydrogen peroxide/peroxidase/luminol system offers a unique and simple tool for quantification of heavy metals. For luminol to exhibit chemiluminescence, an oxidizing agent, an alkaline pH and a catalyst is required. Here, sodium hydroxide provides basic environment, hydrogen peroxide is oxidizing agent, and horseradish peroxidase serves as catalyst (Scheme 1).

Step (1) is catalysed by AlOx which oxidises methanol to formaldehyde and hydrogen peroxide. In step (2) peroxidase converts H_2_O_2_ into H_2_O, O_2_ and light (hυ) is liberated. As a side product aminophtalic acid anion and N_2_ are produced. The signal due to the light intensity (hυ) is correlated to the concentration of methanol and AlOx in solution. In step (3) after the incubation with Hg(II), the enzyme activity was observed in terms of light liberated (hυ). The degree of inhibition was expressed by the ratio of the photon liberated before and after the Hg(II) inhibition.

Individual inhibitions of free AlOx /HRP by Hg(II), Cd(II), and Pb(II) and due to their composite mixtures were determined in 96 as well as 384 micro well plates. Mixtures of three metal ions were prepared by mixing their concentration at their % inhibitory concentration (IC) value. Mixtures were prepared by taking Hg(II) concentration at IC_15_, IC_30,_ and IC_60_ and Cd(II), Pb(II) at IC_15_ and IC_30_ to study their impact in totality. The concentration corresponding to 15% inhibition is written as IC_15_ and same terminology is applied for others. The first row of each plate was used as blank whereas second row was used for the Hg(II) concentration at IC_15_, IC_30,_ & IC_60._ The subsequent rows were kept for the mixture of metals. Various combinations were prepared. The details of each combination are as follows: **Combi 1:**Hg(II) IC_15_ + Cd(II) IC_30_, **Combi 2:** Hg(II) IC_30_ + Cd(II) IC_30_, **Combi 3:** Hg(II) IC_60_ + Cd(II) IC_30,_**Combi 4:** Hg(II) IC_15_ + Pb(II) IC_30_,**Combi 5:** Hg(II) IC_30_ + Pb(II) IC_30_ ,**Combi 6:** Hg(II) IC_60_ + Pb(II) IC_30_,**Combi 7:** Hg(II) IC_15_ + Pb(II) IC_30_ + Cd(II) IC_30_, **Combi 8:** Hg(II) IC_30_ + Pb(II) IC_15_ + Cd(II) IC_15_.

### 2.4. Experimental Data Processing

The degree of inhibition of enzyme activity in the presence and absence of Hg(II) compound can be calculated as follows:

**I% = 100 (hυ_1_ − hυ_2_) / hυ_1_**
**hυ_1_** = Photon count in absence of Hg(II) compound.
**hυ_2_** = Photon count in presence of Hg(II) compound.

Inhibition values were plotted against Hg (II) concentration for dynamic as well as linear range in the calibration curve. The Limit of Detection (LOD) value generally corresponds to 90–80% of residual activity that is 10–20% inhibition. For calculation of detection limit 10–20% of inhibition was taken as a reference [35].

Origin 6.1 (Microcal, USA) was used for processing of data. Experimental data obtained was also treated statistically for regression coefficient, equation for straight line, standard deviation and % CV using the said software.

### 2.5. Sample Preparation

Analysis of real samples requires proper sampling and adequate sample storage facility. Sea water samples were taken during high tide from the inertial region of the Arabian Sea near Goa, India. Sampling was done in sterilized PVC containers. The containers were immediately taken to the laboratory and filtered through 0.45 μm filter paper (Whatman, USA) followed by 0.22 μm filter paper (Millipore, USA) to stop any biological growth and to avoid any interference of suspended particles. Samples were divided into sub samples, transferred into sterilized PVC container and immediately stored at −20 °C. Quite often, natural sea water was collected, filtered, diluted and analyzed on the same day to avoid any microbial growth. Samples were used without any further pretreatment process.

## 3. Results and Discussion

### 3.1. Enzymatic Assay Development: for Bioassay Development the Following Parameters Were Optimized

#### 3.1.1. Effect of Ionic Strength

The effect of ionic strength on the response of bienzyme reaction was studied. PB with varying ionic strength *i.e.*, 0.01–0.2 M was prepared and used as reaction medium. Increased signal intensity was observed with increased ionic strength of PB. Maximum response was observed at 0.1 M ionic strength. As optical detectors are free from any change in the ionic composition of solution, effect of ionic strength on the response of reaction was solely dependent on interaction between AlOx and ions in the reaction medium. The maximum activity of AlOx was observed at 0.1 M ionic strength of PB.

#### 3.1.2. Effect of pH

The effect of pH in 0.1 M PB on the AlOx activity was studied. The activity profile of the enzyme in the pH range 6.8–9 was performed. The maximum enzyme activity was observed at pH 7.5 and as the pH increases further the sharp decrease in activity of about 40% was observed at pH 9. In this study optimum pH, the point where enzyme is most active is observed stable at pH 7.5 and was selected for further studies.

#### 3.1.3. Effect of Enzyme Concentration

**Figure 1.**
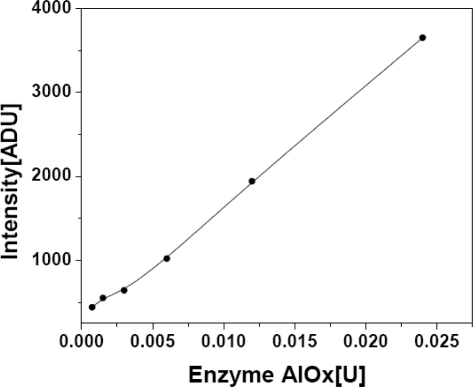
Graph showing the optimization of AlOx concentration for proposed bi-enzyme reaction utilizing AlOx/methanol/HRP/luminol in 96 micro well plate using chemiluminescence techniques.

Enzyme concentration is one of the critical factors to be evaluated in any inhibition based assay. Optimization of reaction parameters for a single enzyme system *i.e.*, HRP/H_2_O_2_ was achieved first and was subsequently optimized for the bi-enzyme reaction. To optimize enzyme concentration, substrate was taken in an excess amount, *i.e.*, reaction is independent of the substrate concentration. Different units of AlOx were allowed to react with 0.5 mM methanol. Enzyme concentrations from 0.00075–0.024U per assay volumes were tested for the optimization of enzyme concentration. Considering the total assay volume *versus* bienzyme response, it was possible to achieve good optical signal with 0.00075U of free AlOx. In the optimized bienzyme reaction, final concentration of AlOx (0.01U) and HRP (0.001U) were used in 100 μL assay. The results are presented in Figure 1.

#### 3.1.4. Effect of Temperature

Like most chemical reactions, the rate of an enzyme-catalyzed reaction increases with an increase in temperature. It is well known that variations in reaction temperature may induce significant changes in enzyme activity. The structure of enzymes is basically affected by temperature fluctuations in the assay. Effect of temperature on the bi-enzyme reaction (AlOx/HRP) was studied by incubating the enzyme at different temperatures ranging from 28–40 °C in micro well plate. The signal intensity was recorded. It was observed that the bienzyme activity increases with the increase in temperature. Optimum activity was observed at 35 °C. Further increase in temperature, resulted in the decrease of bienzyme activity and 20% activity was lost at 40 °C. Thus, further enzyme determinations were carried out at optimum temperature, which was 35 °C.

#### 3.1.5. Optimization of Substrate Specificity and Substrate Concentration

**Figure 2.**
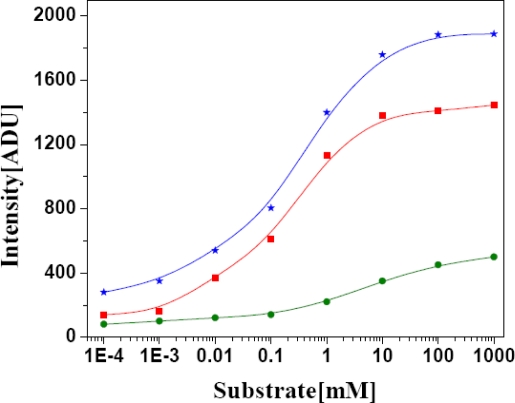
Response curve of the AlOx based assay for substrate determination in the presence of various concentration of substrates, such as methanol, ethanol and propanol in 0.1 M PB pH 7.5 at 35 °C. Response time is 5 min.

For AlOx, various substrates e.g., propanol, ethanol and methanol have been reported. For all the primary alcohols studied in 96 as well as 384 well format, signal intensity increased with increasing substrate concentration, as shown in Figure 2. The signal intensity increases linearly up to 1 mM substrate concentration and remains stable over the range from 0.001–1 M with AlOx in the bi-enzymatic reaction. Among the various substrates, AlOx exhibited highest activity with methanol. Thus methanol was selected for further optimization. In order to determine the K_M_, methanol concentration was varied in the range 1 μM−1 M and response signal against 0.01 U AlOx was recorded. The experimental data was used to calculate K_M_. Alternatively data was fitted with Line weaver Burk plot to reconfirm K_M_ value. The K_M_ for methanol was calculated to be 0.5 mM. Further assays were carried out with 0.5 mM methanol. Optimized assay parameters for enzymatic assay developments are summarized in Table 1.

### 3.2. Inhibition Studies for Heavy Metal Determination

#### 3.2.1. Hg(II) Determination

The optimised experimental parameters were used for inhibition studies in 96 well formats. The difference in bi-enzyme activity before and after exposure to Hg (II) gives a quantitative measure of Hg(II) determination. It is observed that lower percentage of inhibition (higher sensitivity) was achievable using lower AlOx concentrations in the bi-enzyme reactions. To determine low levels of Hg(II) by inhibition, concentration of free AlOx in bi-enzyme reaction was optimised to provide a high sensitivity to inhibition with reasonably good intensity. Inhibition by Hg(II) with different units of AlOx (0.01–0.24 U/assay volume) and 0.5 mM methanol concentration was studied (data not shown).

##### Effect of Incubation Time

**Figure 3.**
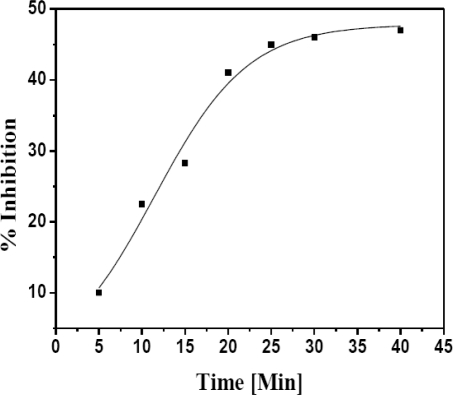
Inhibitory effect of different incubation time for AlOx and Hg(II) obtained at fixed concentration of AlOx (0.01U) and methanol (0.5 mM). The 0.1 mM PB, pH 7.5 was used and reaction was carried out at 35 °C.

Incubation time plays a significant role in the determination of the degree of enzyme inhibition. Wide range of incubation time (2–25 min) is reported for heavy metal determination [9–21]. Incubation time for mixture containing 0.1 ng·mL^−1^ Hg(II) and 0.01 U AlOx was varied in the range 5–40 min. We observed decrease in the enzymatic activity up to 40 min. A linear trend for inhibition with increasing incubation time was observed from 5–20 min with 40% inhibition. From Figure 3 it is clear that after 20 min, further increase in incubation time does not impart any appreciable inhibition to AlOx (47% I). Thus, for further inhibition studies 20 min was optimized as incubation time.

##### Effect of Inhibitor Concentration

For sensitive determination of Hg(II), experiments were conducted for quantitative inhibition by varying Hg(II) concentration from 5 pg·mL^−1^ −50 ng·mL^−1^ using 0.5 mM methanol, 0.01 U AlOx, 0.001 U HRP with 20 min incubation time at 35 °C.

**Figure 4.**
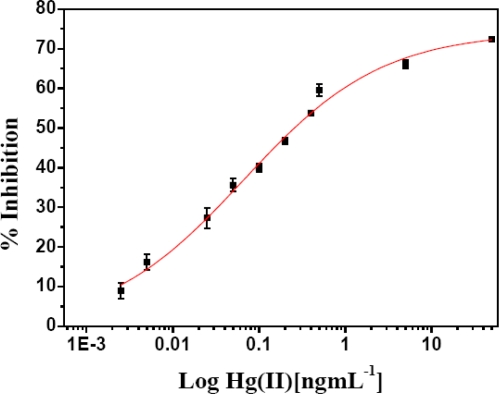
Calibration curve for Hg (II). Percentage of inhibition (% I) *versus* logarithm of Hg(II) concentration in ng·mL^−1^ is presented. The error bar indicates standard deviation (n = 3, where n is an independent assay by proposed method). Degree of inhibition of free AlOx (0.01 U) using 0.5 mM methanol for 20 min incubation time using 0.1 M PB, pH 7.5 at 35 °C. Equation for line is Y = 20.77X + 62.53.

Figure 4 shows the inhibition effect of Hg(II) on free AlOx in bi-enzyme reaction at an optimized contact time of 20 min. Good linearity was observed for low level Hg(II) concentrations in the range 5–500 pg·mL^−1^. Inhibition was achievable proportional to very low concentration of Hg(II) with minimum detection limit of 5 pg·mL^−1^ with coefficient of variation 3% (all measurements were done in triplicate and the data points in graph represent the mean value of the determinations). A much improved linear range and sensitivity of Hg(II) determination is achieved, compared to other reported inhibition biosensors [9–21]. The assay provides additional feature of simultaneously analysing large number of sample. Broad dynamic range from 5 pg·mL^−1^−50 ng·mL^−1^ with high sensitivity up to 2.5 pg·mL^−1^ and R^2^ = 0.9949 are hallmarks of our assay. The equation for line, Y = 20.77 X + 62.53. The figures of merit of inhibition assay parameters are given in Table 2.

#### 3.2.2. Lead and Cadmium Determination

Divalent metal ions other than Hg(II) such as Pb(II), Cd(II) also have affinity to bind with AlOx. Thus, experiments were carried out in order to evaluate their individual and combined inhibition when they are present along with Hg(II).

##### Inhibition by Cd(II) and Pb(II)

**Figure 5.**
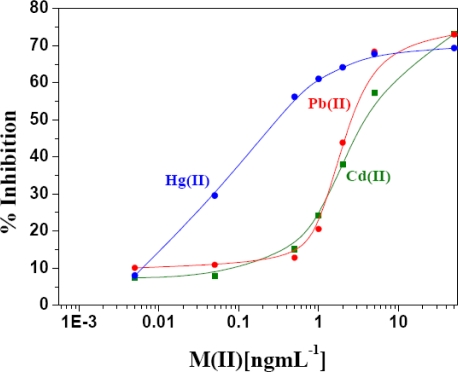
Inhibitory effect of Hg(II), Cd(II) and Pb(II) concentration on AlOx activity at incubation time 20 min. in 96 well plate format.

The specificity of AlOx towards Hg(II), Cd(II) and Pb(II) individually was studied by challenging them with related metal ions. The individual inhibition by metal ions was determined and is presented in Figure 5. Inhibition by three metal ions shows a concentration dependent character. The IC_15_ for Hg(II), Cd(II) and Pb(II) were found to be 0.01320, 0.4794 and 0.6763 ng·mL^−1^, respectively. The inhibition pattern remains same for IC_30_ and IC_60._ These result shows that inhibition by Hg(II) is much more sensitive as compared to Cd(II) and Pb(II) respectively. From inhibition values at various IC_30_ (0.0492, 1.3792, 1.3906 ng·mL^−1^), we can conclude that inhibition of metals follows the order Hg(II) > Cd(II) ≅ Pb(II). These results reconfirm the predominant interaction of Hg(II) as reported in the literature [14,19]. Among the three reported heavy metals, Hg(II) is highly acidic (strong Lewis acid) and thus show highest affinity towards soft base such as –SH group of the enzyme. This can be accounted for the 10-fold higher sensitivity observed for Hg(II) as against Cd(II) and Pb(II). It is clear that within this range Cd(II) and Pb(II) metal ion do not interfere toward recognition of Hg(II). Inhibition of Cd(II) is more sensitive in the lower concentration range whereas for Pb(II) the sensitivity lies in higher range. Inhibition of AlOx by Hg(II) is reversible and competitive involving the -SH group of these protein. The inhibition of AlOx was reversed when a higher concentration of methanol was used.

##### Mixture of Hg(II), Cd(II) and Pb(II)

**Figure 6.**
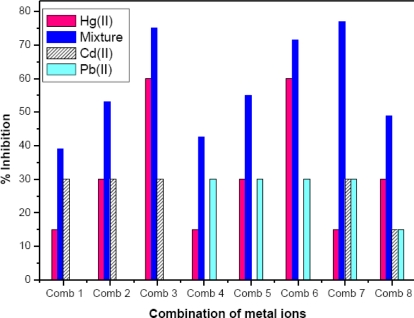
Combined effect of Hg (II), Cd (II) and Pb (II) concentration on enzyme activity at optimized inhibition time. For each combination, concentrations corresponding to IC values are taken. First column corresponds to Hg(II), second to mixture, third column to Cd(II) and fourth to Pb(II). Pb(II) is absent in first three combination, whereas Cd(II) is absent in combination 4, 5 and 6.

Co-existence of Hg(II), Cd(II) and Pb(II) are commonly encountered in food, water and other parts of the environment as a result of human and natural activity. During metal ion analysis, the presence of other metal ion may elicit antagonistic, additive or synergistic effects. In order to understand the toxic effect of these chemicals in totality, it is essential to study the inhibition at various concentration levels. Experiments were carried out to determine relative inhibition of AlOx by binary combination of Hg(II) and Cd(II), Hg(II) and Pb(II) and Hg(II), Cd(II) and Pb(II) and compared these values with the inhibition of Hg(II) alone. In this study, Hg(II) concentrations at IC_15_, IC_30,_ and IC_60_ were mixed with Cd(II) and Pb(II) concentration at IC_30._ When combined with Hg(II) both metal ions exhibit additive effect as depicted in Figure 6. This study on mixture of metal ion can be used as a supporting data for metal induced toxicity by mixture of heavy metal ions using the AlOx bioassay. Recent reports on metal induced toxicological interactions using the MCF 7 cell line [25] and the enzyme urease [19] also support the additive nature of heavy metal ion mixtures, as reported in this work.

Thus, the study demonstrates that all the metal ions inhibit AlOx significantly higher when they are combined than when they inhibit AlOx individually. Various combinations were prepared to study interactive effect. It is evident from Figure 6 that in case of combinations 3 and 6 where Hg(II) concentration is corresponding to IC_60_, the inhibition of AlOx is solely due to Hg(II).

### 3.3. Analysis in Real Samples

**Figure 7.**
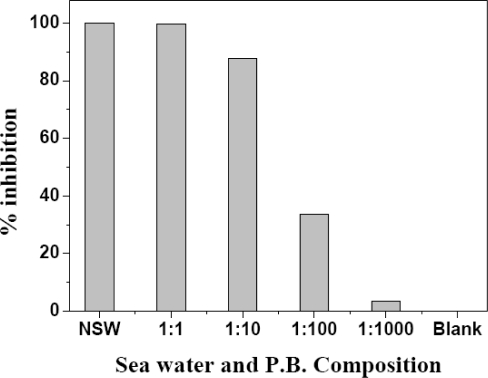
Matrix effect of sea water on enzyme activity. Filtered sample was diluted with 0.1 M PB pH 7.5 in various dilution range and shows minimum matrix effect around 1:1000 dilution where 1 part comprise of sea water and 1,000 part PB *(NSW; natural sea water).*

The Hg(II) concentration in sea water is usually very low. Thus it is very difficult to get precise and reliable analytical results in sea water by direct measurement without separation. The proposed method was used to study the feasibility of Hg(II) analysis in environmental samples such as drinking water, tap water and sea water. For analysis in sea water, fresh and filtered sea water was taken without any acidification. Various dilutions of sea water and buffer were spiked with 75 pg·mL^−1^ of Hg(II) solution. For dilution of sea water sample, dilution with 1,000 parts with 0.1 M PB shows negligible matrix effect, as shown in Figure 7. For determination of Hg(II) in water, the samples were first spiked with 75 pg·mL^−1^ of Hg(II). The results obtained are presented in Table 3. The Hg(II) concentration in spiked real samples was found to be very low with the developed assay. Recoveries in the case of drinking water and tap water were found to 100% and 102.6%, respectively. Sea water shows maximum recovery, up to 110.52%, supporting the presence of Hg(II) in coastal sea water in the studied area (Goa, India). Apart from spiking studies, water samples were analyzed directly without spiking with Hg(II). The results obtained were also matched with conventional ICP-MS results.

### 3.4. Miniaturization of Enzymatic Assay and Highly Sensitive Determination of Hg(II)

The miniaturization of assay provides several advantages such as increased throughput, reduced reagent consumption and reduction in consumables. Reduction in assay volume in 384 well plates provides opportunity for inhibition studies in closer proximity. Thus, inhibition assay presented in 96 well plate format was miniaturized further to 384 well plate format.

#### 3.4.1. Effect of Miniaturization on Substrate Response in 96 and 384 Well Plate Format

Usually, the concentration of reagents needs to be decreased when moving to lower well-density plates due to decreased surface to volume ratio. The role of buffer also increases to minimize non-specific binding. Miniaturization leads to decrease in enzyme per assay requirement. Enzyme consumption as low as 0.002 U with good signal intensity was achieved which is a 5-fold decrease as compared to the 96 well assay. The optimized assay with 5-fold reduction in enzyme and substrate concentration was carried out in 384 well plates and it was observed that K_M_ is independent of miniaturization. About 3-fold higher signal intensity was measured in case of 384 well plates as against 96 well plate assay. This may be due to the close proximity of enzyme and substrate in miniaturized assay format. Close proximity also facilitates mixing of reactants.

#### 3.4.2. Effect of Miniaturization on Inhibition Studies in 96 and 384 Well Plate Format

Miniaturization can dramatically influence incubation time, inhibitor concentration and economy of the assay. Thus, inhibition studies for Hg(II) determination were carried out in 384 well plate format with 20 μL assay volume. A significant 5-fold reduction in volume of Hg(II) solution was achievable. This bears significant positive impact on the environment. Due to the close proximity of enzyme and Hg(II) in the 384 well plate format, incubation time reduces to half. As a result of the miniaturization, lowering of IC_30_ value for Hg(II) from 60 pg·mL^−1^−32 pg·mL^−1^ is also achieved. Low% CV was observed for all assays performed in 384 well plate format, indicating the robustness of the miniaturized assay. Major achievements resulting from miniaturization of assay are summarized in Table 4.

### 3.5. Stability of the Enzyme and Reproducibility of the Assay in Well Plate

**Figure 8.**
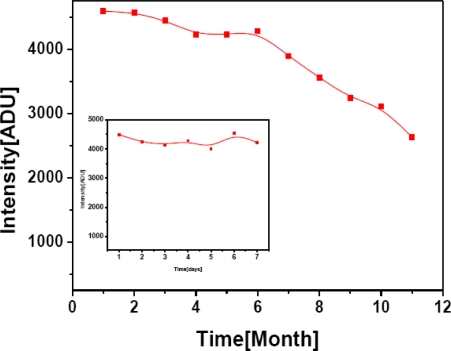
Inter-day stability of AlOx in bi-enzyme reaction in 96 well plate formats activity over the period of 11 months. Inset: activity over the period of one week. 0.5 mM methanol with 0.001U HRP and 1mM luminol were used throughout the assay period.

Stability of bioassay in terms of repetitive uses was performed using 0.5 mM of methanol as substrate over the period of one week initially and then extended to 11 months. Excellent storage stability was observed for stock AlOx in 0.1 M PB at pH 7.5 when AlOx aliquots were preloaded to microwell plate and stored at 4 °C. Relative bioassay responses to 0.5 mM methanol over storage time of 11 months and one week are shown in Figure 8. Each data point corresponds to an average of three measurements and they are plotted relative to original response corresponding to first day. From Figure 8, it is clear that for first three month only 5–6% of enzyme activity was lost, which increased up to 15% during the first eight months. At the end of 11 months the enzyme retained 60% of its activity in free form without any immobilization or stabilization. From the inset figure it is evident that AlOx is a highly stable enzyme and retained its activity (around 95%) when stored at 4 °C in microwell plates over the period of one week. This property can be explored as a tool for high throughput analysis with use of pre-loaded AlOx enzyme.

## 4. Conclusions

In this study the specificity of free AlOx in a bi-enzyme reaction towards Hg(II) was utilized to develop a highly sensitive bioassay for Hg(II) determination in water samples. A chemiluminescence technique was used for quantification of enzyme inhibition. The assay shows a wide dynamic range from 5 pg·mL^−1^ −50 ng·mL^−1^ with linearity in the range 5–500 pg·mL^−1^. Using this assay Hg(II) was determined at levels as low as 5 pg·mL^−1^ which have not been reported till now using free enzyme. The reported results are among the most sensitive values for Hg(II) using free enzyme (AlOx). The assay can determine Hg(II) in presence of Cd(II) and Pb(II). The following ranking for inhibition by metals in the mixture was obtained: Hg(II) > Cd(II) *≅* Pb(II). The IC_15_ for Hg(II), Cd(II) and Pb(II) were found to be 0.01320, 0.4794 and 0.6763 ng·mL^−1^, respectively. When AlOx was exposed to mixtures of metal ions for inhibition, the measured response was found to be additive. The assay can be also used as a toxicity assessment. The applicability of the presented assay was tested by running the assay in real samples. Using mere filtration and dilution of the sample good recoveries (up to 110%) were obtained in spiked samples. AlOx proved to be perfect choice for study due to its high stability in microwell plates over a period of 11 months. The assay was successfully miniaturized in 384 well plate format with an assay volume of 20 μL. IC_30_ values obtained using the 384 well plate format were much lower than those found in 96 well plate format. The LOD was as low as 1 pg·mL^−1^ Hg(II) was achieved. The assay can be easily automated and adopted for high-throughput environmental analysis.

## Figures and Tables

**Scheme 2.**
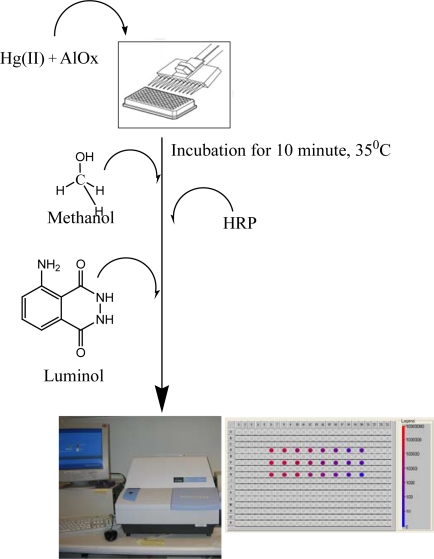
Schematic representation of AlOx assay.

**Table 1.** Optimization of experimental parameters in 96 well plate formats.

**Parameter**	**Experimental range**
Enzyme concentration	0.00075–0.024 U/assay
Substrate specificity	Methanol, ethanol and propanol 10 nM to 1 M
pH of buffer	6.8–9.0
Ionic conductivity of buffer	0.01–0.2 M
Temperature	28–40 °C

**Table 2.** Figures of merit for proposed Hg(II) assay in 96 well plate formats.

**Analyte**	**Hg (II)**
Linear dynamic range	0.005–50 ng·mL^−1^
Analysis time	25 min
Apparent IC_50_	0.3 ng·mL^−1^
R^2^	0.9949
Minimum Detection limit	2.5 pg·mL^−1^
Total assay volume	<100 μL
Throughput	about 70 samples in less than 30 min (leaving blank and reference)

**Table 3.** Recovery studies in real samples using free AlOx/HRP- methanol assay. The assay was carried out in 0.1 M PB, pH 7.5 at 35 °C in 96 well plate formats. 75 pg·mL^−1^of Hg(II) was spiked in real water sample. Column 4 gives the idea of amount of Hg(II) present in the sample.

**Matrix**	**Spiked Hg(II) (pg·mL^−1^)**	**Hg(II) found (pg·mL^−1^)**	**Difference (pg·mL^−1^)**	**% Recovery**
Drinking Water	75	75	-----	100
Tap water	75	77 ± 0.05	2.05	102.66
Sea water-1	75	80 ± 0.75	5.75	106.66
Sea water-2	75	79	4	105.33
Sea water-3	75	82± 0.89	7.89	110.52

**Table 4.** Miniaturization of assay volume and its effect on performance of the bi-enzyme reaction in 96 and 384 micro well plate formats. Inhibition parameters are compared for high throughput analysis.

**Parameter**	**96 well-plate assay**	**384 well plate assay**
Enzyme U/assay	0.01	0.002
Incubation time, min	20	10
IC_30_ (pg·mL^−1^)	60	32
Total assay volume (μL)	100	20
